# Efficacy and Safety of Inhaled Ciclesonide in Treating Patients With Asymptomatic or Mild COVID-19 in the RACCO Trial: Protocol for a Multicenter, Open-label, Randomized Controlled Trial

**DOI:** 10.2196/23830

**Published:** 2020-12-31

**Authors:** Junko Terada-Hirashima, Manabu Suzuki, Yukari Uemura, Masayuki Hojo, Ayako Mikami, Wataru Sugiura, Norio Ohmagari, Haruhito Sugiyama

**Affiliations:** 1 Department of Pulmonary Medicine Center Hospital of the National Center for Global Health and Medicine Tokyo Japan; 2 Center for Clinical Sciences National Center for Global Health and Medicine Tokyo Japan; 3 Disease Control and Prevention Center Center Hospital of the National Center for Global Health and Medicine Tokyo Japan

**Keywords:** COVID-19, SARS-CoV-2, proposed therapy, therapy, drug, treatment, protocol, randomized controlled trial, intervention

## Abstract

**Background:**

Currently, there are no specific effective treatments for SARS-CoV-2 infection; however, various COVID-19 treatment options are under investigation. It is vital to continue investigating the landscape of SARS-CoV-2–induced pneumonia and therapeutic interventions.

**Objective:**

This paper presents the protocol for a randomized controlled trial that aims to compare the pneumonia exacerbation rate between ciclesonide (ALVESCO; Teijin Pharma Limited) administration and symptomatic treatment in patients with COVID-19 and to determine the efficacy of ciclesonide. The secondary objectives are to investigate the safety of ciclesonide administration, changes in clinical and laboratory findings, and the number of viral genome copies of SARS-CoV-2 over time between the 2 groups.

**Methods:**

In this investigator-initiated, exploratory, prospective, multicenter, parallel-group, open-label, randomized controlled trial, a total of 90 patients diagnosed with COVID-19 will be recruited from 21 hospitals in Japan based on specific inclusion and exclusion criteria. Participants will be randomized either to the ciclesonide group, which will receive a 400-µg dose of ciclesonide 3 times per day over a 7-day period, or to the symptomatic treatment group. Both groups will receive antitussives and antipyretics as required. Data collection for various parameters will be conducted on days 1, 2, 4, 8, 22, and 29 to record baseline assessments and the findings over an extended period. Computed tomography images taken prior to drug administration and 1 week following treatment will be compared, and efficacy will be confirmed by checking for pneumonia exacerbation. Primary endpoint analysis will be performed using the Fisher exact test to determine statistically significant differences in the pneumonia exacerbation rate between the ciclesonide and symptomatic treatment groups.

**Results:**

The first trial participant was enrolled on April 3, 2020. Recruitment is expected to be completed on September 30, 2020, while follow-up assessments of all participants are expected to be completed by October 31, 2020. The study results will be published in a peer-reviewed scientific journal.

**Conclusions:**

The RACCO (Randomized Ciclesonid COVID-19) study will provide definitive comparative effectiveness data and important clinical outcomes data between the ciclesonide and symptomatic treatment groups. If the hypotheses that pneumonia exacerbation rate reduction is more significant in the ciclesonide treatment group than in the symptomatic treatment group and that ciclesonide is safe for use are valid, ciclesonide will serve as an important therapeutic option for patients with COVID-19.

**Trial Registration:**

Japan Registry of Clinical Trials jRCTs031190269; https://jrct.niph.go.jp/en-latest-detail/jRCTs031190269

**International Registered Report Identifier (IRRID):**

DERR1-10.2196/23830

## Introduction

### Background and Rationale

An outbreak of severe respiratory illness occurred in Wuhan, China at the end of December 2019. The World Health Organization and China were alerted by an increase in the number of patients with pneumonia of unknown etiology. In January 2020, it was reported that a novel type of coronavirus, SARS-CoV-2, was responsible for the outbreak [1]. Three months later, almost half a million cases of this contagious infection had been identified across 197 countries [2], and on March 11, 2020, the World Health Organization declared the COVID-19 outbreak as a pandemic [3].

While most COVID-19 cases result in mild symptoms, some cases progress to pneumonia, acute respiratory distress syndrome, and death [4,5]. The case fatality rate reported across various countries, settings, and age groups is highly variable, but ranges from less than 1% to 19% [6]. Among hospitalized patients, the case fatality rate has been reported to be greater than 10% in some centers [7].

There is currently no specific effective treatment for SARS-CoV-2 infection, but various options and strategies for treating COVID-19 are under investigation worldwide [8]. Lu et al [9] reported that antiviral molecules, nucleoside analogs, neuraminidase inhibitors, therapeutic peptides, RNA synthesis inhibitors, anti-inflammatory drugs, and Chinese traditional medicine could be therapeutic options for SARS-CoV-2 infection. Currently, the standard treatment methods for clinicians only involve symptomatic treatment; therefore, it is vital to continue investigating the landscape of SARS-CoV-2–induced pneumonia and therapeutic interventions. The asthma drug ciclesonide (ALVESCO) is a pressurized metered-dose inhaler that uses the glucocorticoid ciclesonide and originates from Nycomed. It is now developed, manufactured, and marketed by numerous pharma companies worldwide, including Covis Pharma, Takeda, Sunovion Pharmaceutical, and Teijin Pharma [10]. Teijin Pharma in Japan developed ciclesonide for adult bronchial asthma in April 2007, and it was approved for child doses in January 2011. This drug acts by reducing the swelling of the airways in the lungs in order to make breathing easier. After the oral inhalation of ciclesonide, a prodrug is enzymatically hydrolyzed in the lungs to an active metabolite (ie, des-ciclesonide). Des-ciclesonide has an anti-inflammatory effect and binds to glucocorticoid receptors, thereby controlling chronic inflammation in the air ducts [11].

Preclinical data [12] have suggested that ciclesonide could potentially treat COVID-19, since it has been shown to suppress the replication of human coronaviruses (eg, Middle East respiratory syndrome–related coronavirus) in cell cultures [13] and exhibit strong antiviral activity, even against SARS-CoV-2 [12]. Additionally, a recently published case series [14,15] showed that treatment with this drug has led to favorable outcomes in 3 patients with confirmed COVID-19 [14,15]. These case reports from Iwabuchi et al [14,15] indicated that the ideal administration period should be in the early intermediate stage of infection or in the initial stage of pneumonia, prior to the worsening of symptoms. Covis Pharma has launched a phase III clinical trial in the United States to evaluate its asthma drug, ciclesonide (ALVESCO), for its ability to treat nonhospitalized, symptomatic patients with COVID-19 aged ≥12 years. As of May 22, 2020, the drug is currently being investigated for the treatment of COVID-19 in several countries, such as Australia, South Korea, Sweden, the United Kingdom, and the United States [10], and the results are not yet available.

More studies on ciclesonide are required to assess its efficacy and safety in treating patients with COVID-19. This prospective randomized controlled trial in Japan aims to investigate the efficacy and safety of ciclesonide in treating patients with COVID-19 during the early stages of SARS-CoV-2 infection. The accumulation of findings from this study will be significant for the treatment of COVID-19.

### Objectives

The primary objective of the RACCO (Randomized Ciclesonide COVID-19) trial is to compare the pneumonia exacerbation rate between the ciclesonide and symptomatic treatment groups, which consist of patients with COVID-19, and to determine the efficacy of ciclesonide. The secondary objective is to investigate the safety of ciclesonide administration by comparing the rate of adverse events between both groups. In addition, changes in clinical and laboratory findings and changes in the number of copies of the viral genome over time will be monitored between the 2 treatment groups.

### Trial Design

**Figure 1:**
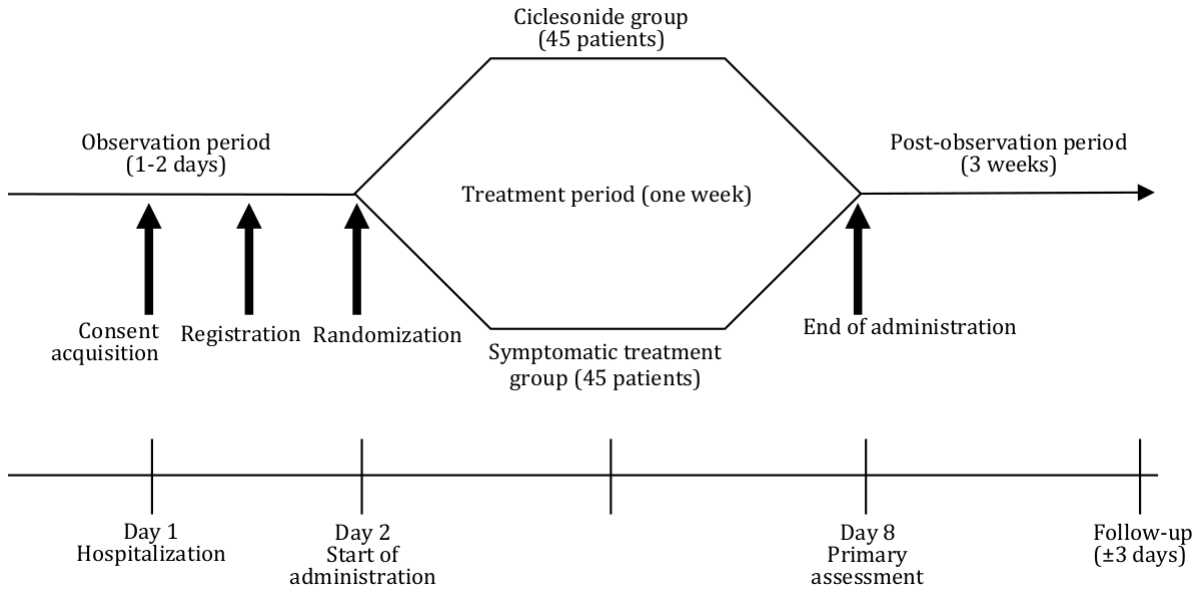
Trial design and outline.

This is an investigator-initiated, exploratory, prospective, multicenter, parallel-group, open-label, randomized controlled trial. Figure 1 describes and outlines the design in more detail. The first trial participant was enrolled on April 3, 2020. Recruitment is expected to be completed on September 30, 2020, while the follow-up of all participants is expected to be completed by October 31, 2020.

## Methods

### Ethical Approval

This study is being conducted in compliance with the Declaration of Helsinki 2013; Clinical Trials Act; Clinical Trials Act Enforcement Regulations; 2018 Ordinance of the Japanese Ministry of Health, Labor, and Welfare; and Japanese Good Clinical Practice (GCP). In the Japanese GCP, principal investigators in all participating institutions serve as the study sponsor for investigator-initiated, registration-directed trials, and hospital directors take responsibility for the study conduct. Prior ethical approval for the protocol and the informed consent document was obtained from The University of Tokyo, Clinical Research Review Board (Approval No CRB3180024), and each participating hospital investigator received permission from the administrator to conduct the study, after which the representative physician submitted the explanatory and consent documents to the Ministry of Health, Labor, and Welfare in Japan. This trial is registered and recorded in the Japan Registry of Clinical Trials (jRCT; jRCTs031190269), and research only commenced after the jRCT release.

### Study Setting

The main study site is the Center Hospital of the National Center for Global Health and Medicine, Tokyo, Japan, but several other hospitals are participating in this trial. A total of 90 patients diagnosed with COVID-19 were recruited from 22 hospitals in Japan. Textbox 1 lists the medical institutions participating in this trial.

The sample size for the RACCO trial (N=90) is based on a statistical minimum number of patients required for each treatment arm, to control for pneumonia onset and the final number of patients who will undergo the full analysis and complete the study. The sample size calculation is outlined under the Statistical Methods section.

**Participating medical institutions.**

**Names of the implementing medical institutions**

- Center Hospital of the National Center for Global Health and Medicine
- The University of Tokyo Hospital
- Toho University Omori Medical Center
- Showa University Hospital
- International University of Health and Welfare Narita Hospital
- International University of Health and Welfare Mita Hospital
- Atsugi City Hospital
- National Hospital Organization Higashisaga Hospital
- National Hospital Organization Numata Hospital
- Iizuka Hospital
- National Hospital Organization Nishisaitama-chuo National Hospital
- Daini Osaka Police Hospital
- National Hospital Organization Nara Medical Center
- Sapporo City General Hospital
- National Hospital Organization Kokura Medical Center
- Yoshida Hospital
- Ome Municipal General Hospital
- National Hospital Organization Kyushu Medical Center
- National Hospital Organization Fukuyama Medical Center
- Kanagawa Prefectural Hospital Organization Kanagawa Cardiovascular and Respiratory Center
- National Hospital Organization Osaka National Hospital
- The Fraternity Memorial Hospital

### Recruitment

Strategies have been implemented to ensure maximum enrollment at each site. Each study site has a target number of patients to enroll. Furthermore, our study period includes the following: the registration period, which lasts from March 30 to September 30, 2020; the observation period, which lasts from March 30 to October 31, 2020; and the implementation period, which lasts from March 30 to October 31, 2021.

### Eligibility Criteria

Our inclusion criteria for study participants are as follows: (1) provided written consent for research participation; (2) aged ≥20 years at the time of consent acquisition, regardless of sex; (3) tested positive for SARS-CoV-2 based on polymerase chain reaction (PCR) results or the loop-mediated isothermal amplification method (4) had no clear indications of COVID-19–induced pneumonia on simple chest images; (5) hospitalized during the trial drug administration period (ie, 1 week); and (6) had the ability to receive ciclesonide inhalation using an inhalation-assisting device.

Our exclusion criteria for study participants are as follows: (1) a medical history of ciclesonide hypersensitivity; (2) the presence of an infectious disease or deep-seated mycosis other than COVID-19, for which there is no effective antibacterial agent; (3) the presence of chronic respiratory diseases, such as chronic bronchitis; (4) current treatment with inhaled or oral steroids; (5) a history of a continuous fever of ≥37.5°C that lasted for over 7 days; (6) current treatment with agents that have potential therapeutic effects against COVID-19 and may affect the efficacy assessments, including remdesivir, lopinavir/ritonavir compound drugs, favipiravir, interferon, and hydroxychloroquine; and (7) conditions deemed unsuitable for research by the principal physician or subphysician.

### Informed Consent

Physicians trained in GCP will determine participants’ eligibility, discuss the trial with the patients, and seek their informed consent. Explanatory and consent documents approved by the authorizing clinical study examination committee and authorized by the Minister of Health, Labor, and Welfare will be provided to the research participants, and sufficient written and verbal explanations will be provided in order to avoid forceful or unfair influences. Consent from research participants will be obtained by having them sign and date the consent document.

### Patient Registration and Allocation Method

After obtaining written consent from the research participants, the principal physician or subphysician will confirm whether a research participant is suitable for the trial by verifying the inclusion and exclusion criteria, promptly performing electronic data capture (EDC)-based patient registration, and acquiring the allocation results.

A stratified block randomization method will be used to allocate participants into 2 groups, the ciclesonide group and symptomatic treatment group. Allocation adjustment factors include the numbers allocated to each facility, fever temperature (ie, over or below 37.5°C), and age (ie, over or under 60 years). Allocation results will be displayed on the EDC system after inputting and confirming the allocation adjustment factors into the EDC system.

The principal physician or subphysician will commence research treatment in accordance with the allocation results. Research participants will be issued a research ID that is specific to the proposed research.

### Interventions

Participants will be randomized to 1 of the 2 treatment groups. In the ciclesonide group, a 400-µg dose of ALVESCO, which is manufactured by Teijin Pharma Limited, Japan, will be administered to patients with asymptomatic or mild COVID-19 3 times per day over a continuous 7-day period. The patients will also receive antitussives and antipyretics as needed. In the symptomatic treatment group, only antitussives and antipyretics will be administered to the patients as needed.

Ciclesonide has not been approved for COVID-19 treatment in Japan or internationally, and is used only for research purposes.

### Criteria for the Discontinuation of Treatment/Intervention

The representative physician will review the continuation of research implementation when the following events occur: (1) when important information relating to the trial drug quality, efficacy, and safety; factors that may affect the research implementation or continuation; and other factors that affect the effective implementation of the research is obtained; (2) when research subject incorporation and the attainment of the predicted number of participants becomes difficult; (3) when research objectives are achieved during intermediate analysis prior to reaching the predicted number of participants or completion of the predicted period; (4) when the authorizing clinical study examination committee instructs a revision of the research proposal and when this revision is difficult to incorporate; (5) when the authorizing clinical study examination committee issues a suspension; and (6) when serious or continuous violations of the clinical research methods, enforcement regulations, or this research proposal are noted.

### Strategies to Improve Adherence to Intervention

A face-to-face initiation meeting was held prior to the commencement of the trial at each site. Site investigators and research staff engaged with their multidisciplinary teams and provided training.

The principal physician or subphysician will provide guidance for research participants prior to the start of research. Research participants will be shown an inhalation guidance video beforehand and will be guided on how to use the AeroChamber Plus (Trudell Medical UK Limited) in order to increase respiratory efficiency. Furthermore, an asthma diary will be distributed to the patients in advance. Patients are to confirm that the inhalant has not yet been administered, and the patient will be instructed to check the confirmation column after inhalation.

### Relevant Concomitant Care Permitted or Prohibited During the Trial

The following drugs are prohibited during the research implementation period (ie, from consent acquisition to the final observation date): (1) orally or intravenously administered systemic adrenocortical steroids, (2) inhaled steroids other than inhaled ciclesonide, and (3) drugs that may have therapeutic effects against COVID-19 (eg, remdesivir, lopinavir/ritonavir compound drugs, favipiravir, interferon, and hydroxychloroquine).

### Provisions of Posttrial Care

The expected research participation period will last for approximately 1 month, after consent acquisition (ie, 1 week for the protocol treatment period and 3 weeks for the follow-up period). Research participants will be hospitalized during the treatment period, and outpatient follow-up observations will be permitted following the end of the protocol treatment period. In cases where the research participants experience adverse events linked to the treatment, follow-up examinations are to be conducted until the principal physician or subphysician has ensured the safety of the research subject.

### Outcomes

#### Primary Outcome Measure

The primary outcome is the pneumonia exacerbation rate within 7 days after ciclesonide inhalation. Computed tomography (CT) images taken prior to drug administration and 1 week following treatment will be compared, and efficacy will be confirmed by checking for pneumonia exacerbation. Pneumonia exacerbation will be independently diagnosed by 2 radiologists, who will only refer to the CT images. An exacerbation of pneumonia will be diagnosed if evidence of exacerbation is present on the day 8 CT image compared to the day 1 CT image.

#### Secondary Outcome Measures

Secondary outcome measures include changes in clinical findings, laboratory findings, and the number of quantified SARS-CoV-2 viral genome copies over time. Clinical findings include the following: body temperature, malaise/appetite, oxygen therapy conditions, respiration rate, oxygen saturation, systolic blood pressure, heart rate, level of consciousness, hospital discharge, ventilator use, extracorporeal membrane oxygenation use, presence of intensive care unit management, and miscellaneous combined medicine conditions.

Laboratory findings include the following: albumin level, lymphocyte count, C-reactive protein level, D-dimer level, leukocyte count, hemoglobin level, platelet count, blood sedimentation, bilirubin level, aspartate transaminase level, alanine transaminase level, lactate dehydrogenase level, γ-glutamyl transpeptidase level, alkaline phosphatase level, creatine kinase level, creatinine level, blood urea nitrogen level, sodium level, potassium level, glutamic acid level, and procalcitonin level.

The safety endpoint will be based on adverse event frequency/percentage.

### Participant Timeline

**Figure 2:**
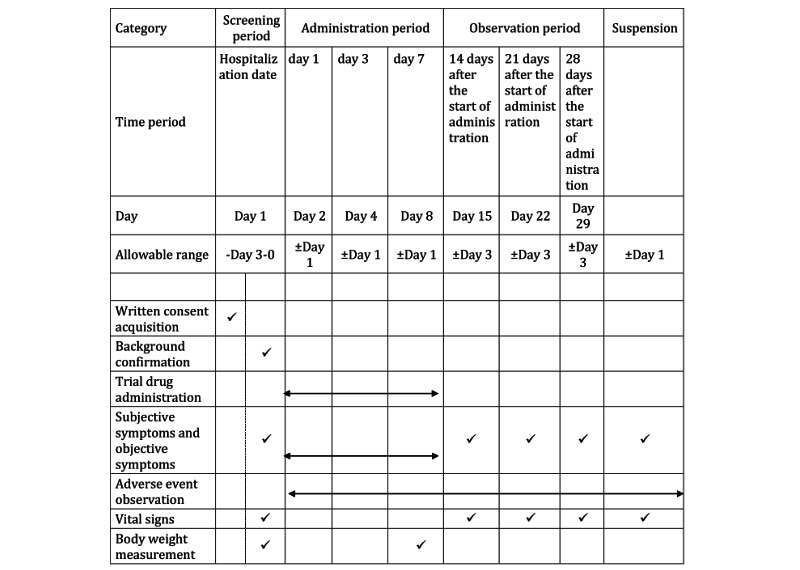
Participant timeline, enrollment, interventions, and assessments.

Figure 2 and Table 1 show the schedule of enrollment, interventions, and assessments. Data collection will occur at screening (ie, day 1), day 2 (ie, randomization period), and days 4, 8, 15, 22, and 29. 

**Table 1:** Assessment details and days of implementation.

Assessment	Screening period	Administration period	Observation period	Suspension
Hematologic test^a^	Day 1	Days 4 and 8	Day 15: implemented as an outpatient examination when the research subject has been discharged. Days 22 and 29: unnecessary when the research subject has been discharged.	Unnecessary when the research subject has been discharged
Biochemical blood inspection^b^	Day 1	Days 4 and 8	Day 15: implemented as an outpatient examination when the research subject has been discharged. Days 22 and 29: unnecessary when the research subject has been discharged.	Unnecessary when the research subject has been discharged
Infectious disease inspection^c^	Day 1	Not implemented	Not implemented	Not implemented
Coagulation test^d^	Day 1	Days 4 and 8	Day 15: implemented as an outpatient examination when the research subject has been discharged. Days 22 and 29: unnecessary when the research subject has been discharged.	Unnecessary when the research subject has been discharged
Coronavirus inspection^e^	Day 1	Day 8	Day 15: implemented as an outpatient examination when the research subject has been discharged.	Implemented when suspended before Day 8
Image inspection^f^	Day 1	Day 8	Day 15 Days 22 and 29: unnecessary when the research subject has been discharged.	Implemented when suspended before Day 8
Peak flow measurement^g^	Day 1	Performed daily	Days 15, 22, and 29	Not implemented
Questionnaire^h^	Day 1	Subjective symptoms recorded daily	Day 15: implemented as an outpatient examination when the research subject has been discharged. Days 22 and 19: obtained by phone when the research subject has been discharged.	Obtained by phone when the research subject has been discharged
Comprehensive assessment by the physician	Day 1	Day 8	Day 15: implemented as an outpatient examination when the research subject has been discharged. Days 22 and 29: unnecessary when the research subject has been discharged.	Unnecessary when the research subject has been discharged
Confirmation of combined drug usage conditions^i^	Not implemented	Information on the presence of concomitant medications collected daily	Days 15, 22, and 29	Not implemented

^a^Leukocyte count, neutrophil count, lymphocyte count, hematocrit level, hemoglobin level, platelet count, and blood sedimentation are to be measured for the hematologic tests.

^b^Albumin, bilirubin, aspartate transaminase, alanine transaminase, lactate dehydrogenase, γ-glutamyl transpeptidase, alkaline phosphatase, creatinine kinase, creatinine, blood urea nitrogen, sodium, potassium, C-reactive protein, glutamic acid, and procalcitonin levels are to be measured for biochemical blood inspections.

^c^Hepatitis B surface antigens, hepatitis B core antibodies, hepatitis C virus antibodies, and HIV antibodies are to be measured for infectious disease inspections.

^d^Prothrombin time, activated partial thromboplastin time, fibrinogen level, and D-dimer level are to be measured for coagulation tests.

^e^For coronavirus inspections, SARS-CoV-2 real-time polymerase chain reaction samples are to be collected with nasal swabs and measured on the day of and 8 days after hospitalization, and serum SARS-CoV-2 antibodies are to be measured on the day of and 8 days after hospitalization.

^f^Chest computed tomography scans are to be taken on the day of and 8 days after hospitalization, and simple chest radiographs are to be taken 4, 8, 15, 22, and 29 days after hospitalization. In addition, image inspections will be conducted as necessary when symptom exacerbation is suspected. Simple chest radiographs on days 22 and 29 will not be taken if the research subject is already discharged. Computed tomography is to be performed when pneumonia worsens within 8 days of hospitalization and when the research is suspended.

^g^Peak expiratory flow is to be measured every morning with a peak flow meter during hospitalization and recorded in a diary.

^h^To be conducted prior to assessment by the physician (eg, before the examination).

^i^Information on drug and treatments other than the trial drug are to be collected.

### Data Collection Items and Management

With regard to research subject demographics and background medical information, the following demographic and medical data will be collected: sex, birth date (ie, age), initials, nationality, ethnicity, height, smoking history (ie, presence/absence, the number of cigarettes per day, and the number of years of smoking), complications, medical history, history of present illness, drug allergies, COVID-19 onset date, PCR positivity date, level of consciousness, hospitalization conditions, ventilator use, extracorporeal membrane oxygenation use, oxygen therapy conditions, physical activity conditions, the presence of intensive care unit management, and miscellaneous combined drug conditions. With regard to subjective and objective symptoms, data on physical findings confirmed through visual, tactile, auditory, and percussion inspections will be collected.

With regard to adverse event observation, in cases where adverse events are confirmed in the research subject, the following information will be confirmed, and the content will be recorded in the clinical record and case report: adverse event name, extent, severity assessment, causal relationship, onset time, outcome, and elimination period.

With regard to vital signs, the following data will be collected during resting conditions: blood pressure, pulse rate, respiratory rate, body temperature, and oxygen saturation. In terms of body weight measurements, body weight and body mass index will be recorded.

Details for blood sample collection are found in Textbox 2.

**Blood sample collection and analysis.**

**Hematologic test**

- The following were measured at each implementing institution: leukocyte count, neutrophil count, lymphocyte count, hematocrit level, hemoglobin level, platelet count, and blood sedimentation.

**Biochemical blood inspection**

- The following were measured at each implementing institution: albumin level, bilirubin level, aspartate transaminase level, alanine transaminase level, lactate dehydrogenase level, γ-glutamyl transpeptidase level, alkaline phosphate level, creatinine kinase level, creatine level, blood urea nitrogen level, sodium level, potassium level, C-reactive protein level, glutamic acid level, and procalcitonin level.

**Infectious disease inspection**

- The following were measured at each implementing institution: hepatitis B surface antigen level, hepatitis B core antibody level, hepatitis C virus antibody level, and HIV antibody level.

**Coagulation test**

- The following were measured at each implementing medical institution: prothrombin time, activated partial thromboplastin time, fibrinogen level, and D-dimer level.

**Coronavirus inspection**

- Nasal swabs will be taken to test for SARS-CoV-2 infection via real-time polymerase chain reaction. Samples will be temporarily stored at each participating medical institution (ie, stored at below −80°C for blood serum), collected by the National Center for Global Health and Medicine, and quantified at the Tokyo Metropolitan Institute of Public Health.
- Serum samples will be taken to test for the presence of SARS-CoV-2 antibodies. Samples will be temporarily stored at each implementing medical institution (ie, stored at below −80°C for blood serum) and quantified at the National Center for Global Health and Medicine.

The following will be performed for image inspection: high-resolution CT on days 1 and 8, and chest radiography on days 1, 8, 15, 22, and 29.

With regard to peak expiratory flow, measurements will be performed every morning with a peak flow meter and recorded in a diary.

A questionnaire assessment using a 10-stage assessment scale for appetite, fatigue, and cough will be issued on days 1-8, 15, 22, and 29. This questionnaire is to be administered prior to the physician-based assessment (eg, diagnosis) to eliminate bias. The subphysician is not to behave in a manner that induces answers from respondents, and the respondent who is handed the questionnaire must respond to it individually.

A comprehensive assessment is to be conducted by the physician. For this assessment, a 3-stage assessment for general patient conditions is to be conducted on days 8 and 15, relative to day 1. The confirmation of combined drug use conditions will begin on the start date of drug administration and continue until the administration period is complete. Trial drug/combined drug administration conditions will also be recorded.

Discharge outcomes are to be recorded.

Data input into the case report and inquiry responses are to be conducted through the EDC system (REDCap [Research Electronic Data Capture]). A manual with details on inputting data, such as input methods and duration, has been created. Chest CT images and simple chest radiographs taken when research is suspended due to pneumonia onset within 7 days of administration will be sent to the Center Hospital of the National Center for Global Health and Medicine by CD-ROM.

### Adverse Event Reporting

The incidence of adverse events, including abnormal variations in clinical inspection values and physiological function inspections, will be recorded. Adverse event incidence duration/elimination duration, extent, treatment, outcomes, severity assessments, correlation with medicinal drugs, and predictability are to be recorded in the diagnostic record and case report.

Adverse event extent will be determined based on certain criteria. Mild adverse events refer to conditions in which continued drug administration is possible with no treatment. Moderate adverse events refer to conditions in which continued drug administration is possible with some form of treatment. Severe adverse events refer to conditions in which drug administration is or should be suspended.

Adverse event severity will be determined using the following criteria: (1) death, (2) conditions that may lead to death, (3) conditions for which hospitalization at a medical institution is necessary or hospitalization duration must be extended for treatment (4) disorders, (5) conditions that may lead to disorders, (6) conditions that are severe according to criteria 1-5, and (7) congenital illnesses or abnormalities due to prior generations. All conditions that are not considered severe will be categorized as nonsevere.

### Serious Adverse Events and Disease Reporting

The following procedures will be undertaken when illnesses occur in the present research. With regard to illnesses, this study will use certain definitions relating to disease.

- The term “disease” was defined as an illness, disability, mortality, or infectious disease caused by the implementation of the clinical research, as well as clinical inspection value abnormalities or symptoms. “Disease” was also defined as adverse events that have a causal relationship with the proposed research that cannot be refuted.
- The term “predictability” refers to diseases with incidence tendencies (eg, research subject drug-based disease incidence, the number of incidents, incidence frequency, and incidence conditions) in the disease report that can be predicted from research proposals or documents, explanations, and attached documents that include a summary of the drug. Incidence tendencies that can be predicted will be indicated as “known,” and those that cannot be predicted will be indicated as “unknown.” As a general principle, drugs that have already been approved for research subjects will use the attached documentation as the decision criteria.
- The term “causal relationship” refers to diseases that have a causal relationship with the proposed research that cannot be refuted. At the very least, the correlation with the research subject drug will be determined as a logical possibility, using the GCP ordinance as a guide.

During the implementation of the proposed research, adverse events will be reported in accordance with national guidelines.

### Plans for the Assessment and Collection of Outcomes

Participants will be assessed, enrolled, randomized, and followed up. Clinical and laboratory data will be collected by study staff.

### Plans to Promote Participant Retention

It has been anticipated that participants will be in the hospital during the treatment phase. Subsequent outpatient visits to the study site will be performed and timed with routine clinic appointments, as per the protocol.

### Data Management

The EDC system, REDCap, will be used for the collection of trial data. An input manual with details on the input methodology will be created. Chest CT images and chest radiographs taken when the research is suspended due to pneumonia onset are to be sent to the Center Hospital of the National Center for Global Health and Medicine by CD-ROM.

### Confidentiality

Any information that may identify a participant will be excluded from publicly presented data. Furthermore, all study-related information will be stored securely at each study site. All copies of clinical records, reports, data collection documentation, process documentation, and administrative forms will be identified through coded identification. Additionally, the principal physician will retain the stored records that relate to the proposed research for 5 years following the day the proposed research is completed.

### Storage of Biological Specimens

Serum and nasal swabs are to be stored in a freezer at a temperature of below −80°C.

The samples from the collaborating research implementing medical institutions will be sent to the National Center for Global Health and Medicine. Serum SARS-CoV-2 antibody values will be measured at the National Center for Global Health and Medicine laboratory, and SARS-CoV-2 PCR assays will be performed at the Tokyo Metropolitan Institute of Public Health. All samples will be stored for 5 years following the day the proposed research is completed.

### Statistical Methods

#### Sample Size

The sample size for the RACCO trial will be 90 patients (ie, n=45 for the ciclesonide group and n=45 patients for the symptomatic treatment group) to control for pneumonia onset and allow for the full analysis of study participants to complete the study.

**Figure 3:**
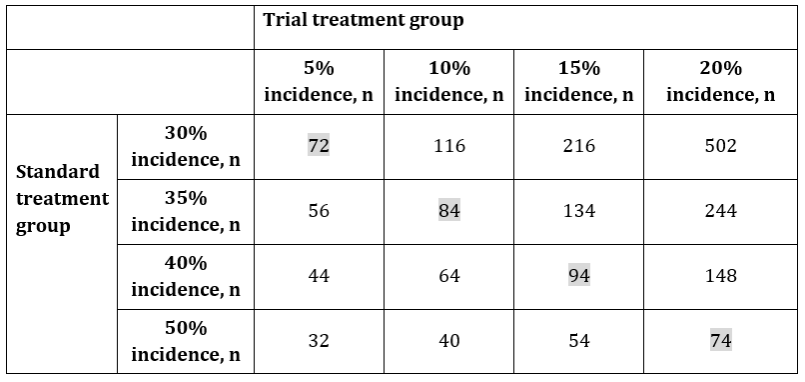
Sample size calculation. Numbers highlighted in grey represent the sample sizes necessary for detecting effects with a power of 80% in cases where a 25% pneumonia onset control is present.

Based on our experience, 35% of patients who have tested positive for COVID-19 without pneumonia symptoms will have pneumonia during the follow-up period. The necessary number of cases for a 2-sided α-level of 10% and power of 80% for cases with incidence percentages of 30%, 35%, 40%, and 50% in the standard treatment group and incidence percentages of 5%, 10%, 15%, and 20% in the trial treatment group are shown in Figure 3.

The effect of controlling the pneumonia onset percentage to 25% would mean that the drug is clinically effective. The numbers highlighted in grey in Figure 3 are the sample sizes necessary for detecting effects with a power of 80% in cases where a 25% pneumonia onset control is present. For example, if the pneumonia onset percentage of the standard treatment and trial treatment groups were hypothetically set to 35% and 10%, respectively, the required sample size would be 84. Factoring in cases where consent may be withdrawn during the trial or participants drop out, the target number of cases for the present research will be set at N=90. The target number of cases will be specified for each implementing medical institution.

#### Analysis Sets

This study will contain the following 3 analysis sets: the full analysis set, the per protocol set, and the safety analysis set. The full analysis set has been defined as research participants who have been registered in the proposed research, have been randomized, have had their baseline CT image data acquired, and have no serious protocol violations.

The per protocol set has been defined as research participants from the full analysis set after removing cases with the following serious protocol violations: selection criteria violations, exclusion criteria violations, prohibited drug violations, prohibited treatment violations, and a medicine administration compliance of below 90%.

In the safety analysis set, all participants will be analyzed.

#### Statistical Methods for Primary and Secondary Outcomes

Analyses are to be conducted after drug administration to all patients has been completed and the data have been finalized. For all efficacy assessments, the full analysis set analysis will be set as the primary analysis, and the per protocol set analysis will be conducted as a reference. A safety analysis will also be conducted using the safety analysis set.

The distribution of research subject background data and the summary statistics in each analysis set will be calculated for each group. For nominal variables, category frequency and percentage will be shown for each group. For continuous variables, the summary statistics (ie, number of cases, average value, standard deviation, minimum value, median value, and maximum value) will be calculated for each group.

Intergroup comparisons will be performed with the Pearson Chi-squared test or Fisher exact test for nominal variables. The 2-tailed Student *t* test or Wilcoxon signed-rank test will be used for continuous variables. The significance level will be set at 10% for both sides.

#### Primary Endpoint Analysis

The primary objective of this study is to verify if the pneumonia exacerbation rate outcome on day 8 in patients with COVID-19 in the ciclesonide administration group is significantly increased compared to that of the symptomatic treatment group.

The primary endpoint analysis will be performed using the Fisher exact test to determine statistically significant differences in pneumonia exacerbation percentages between the ciclesonide and symptomatic treatment groups. The 90% and 95% confidence intervals will be calculated, along with the risk ratio. Secondary analysis will involve identical analyses on the group of patients who do not show evidence of clear pneumonia on simple chest radiographs, but have evidence of pneumonia on chest CT images, and another group of patients whose pneumonia was not observed on chest CT images.

A subgroup analysis based on the following classifications will be conducted: fever presence (ie, over or below 37.5°C), age (ie, over or under 60 years), smoking history (ie, present or absent), and implementing medical institution.

The significance level will be set at 10% for both sides.

#### Secondary Endpoint Analysis

A secondary endpoint analysis will be conducted with the aim of supplementing the primary analysis results. The significance level of hypothesis testing will be set at 5% for both sides, and the 95% confidence interval for both sides will be calculated.

##### Pneumonia Exacerbation

The number and percentage of cases divided among 3 assessment levels, including remission or stabilization cases, potential exacerbation cases, and clear exacerbation cases, will be calculated, and intergroup comparisons will be conducted using the Mantel test.

##### Change in the Quantity of the Virus Genomes

Quantified virus genomes at each assessment time point, quantified antibody values, and related changes will be calculated for each group and illustrated over time. Furthermore, the average difference between groups and the 95% confidence intervals will be calculated, and intergroup comparisons will be conducted.

##### Body Temperature, Respiratory Rate, Oxygen Saturation, Systolic Blood Pressure, and Pulse Rate

The recorded metrics of each assessment criterion at each assessment time point will be calculated for each group, and changes over time will be illustrated. The least mean square and 95% confidence interval at each assessment time point, as well as the least mean square difference and 95% confidence interval, will be calculated. The amount of change from day 1 will be used as the result variable by using mixed models for repeated measures (MMRMs), and intergroup comparisons will be conducted.

##### Fever Duration

The recorded fever temperature for the number of days in which a fever of at least 37.5°C was present during the trial period will be calculated, and intergroup comparisons will be conducted using a 2-tailed *t* test.

##### Symptom Changes

Recorded symptom scores (ie, malaise/appetite scores) at each time point will be calculated, and changes over time will be illustrated. The least mean square and 95% confidence interval at each assessment time point, as well as the least mean square difference and 95% confidence interval, will be calculated. The amount of change from day 1 will be used as the result variable by using MMRMs, and intergroup comparisons will be conducted.

##### Changes in Inspection Findings

Recorded metrics for the continuous values obtained through clinical inspection will be calculated at each time point. Furthermore, the least mean square and 95% confidence interval at each assessment time point, as well as the least mean square difference and 95% confidence interval, will be calculated. The amount of change from day 1 will be used as the result variable by using MMRMs, and intergroup comparisons will be conducted.

For categorical variables obtained from inspection findings, the number and percentage of each criterion at each time point will be calculated, and intergroup comparisons will be conducted using a Mantel test.

##### Oxygen Therapy

The number and percentage of research participants who underwent oxygen therapy during the trial period will be calculated for each group and compared using the Fisher exact test. Furthermore, the average oxygen therapy duration and its 95% confidence interval will be calculated for each group, and intergroup comparisons will be conducted using a 2-tailed *t* test.

##### Hospital Discharge

The number and percentage of research participants who are discharged from the hospital will be calculated for each group, and comparisons will be performed using the Fisher exact test. Furthermore, a Kaplan-Meier curve for the time until hospital discharge will be calculated.

##### Peak Expiratory Flow Measurement

Recorded statistics for peak expiratory flow at each time point will be calculated for each group, and changes over time will be illustrated. Furthermore, the least mean square and 95% confidence interval at each assessment time point, as well as the least mean square difference and 95% confidence interval, will be calculated. The amount of change from day 1 will be used as the result variable by using MMRMs, and intergroup comparisons will be conducted.

##### Comprehensive Assessments by Physicians

The number and percentage of research participants at the 3 comprehensive assessment levels will be calculated for days 8 and 15, and intergroup comparisons will be conducted using the Fisher exact test.

#### Safety Analysis

The safety endpoints include adverse event incidence and rates. The 95% confidence intervals for determining binomial distribution accuracy will be calculated for the estimated rates that relate to the incidence number and presence of adverse events. Intergroup comparisons will be conducted using the Pearson Chi-squared test or Fisher exact test. The significance level of hypothesis testing will be set at 5% for both sides.

Recorded metrics that are calculated for continuous data, such as clinical inspection criteria and vital signs, and suitable methods (eg, number and percentage) will be used as categorical variables for each group.

#### Interim Analysis

An intermediate analysis will be conducted after 50% participant enrollment has been achieved to determine whether the primary objective has been attained. If the trial treatment group shows significantly higher efficacy results than the standard treatment group (ie, efficacy suspension), or if the trial treatment group shows significantly lower efficacy results than the standard treatment group and drug superiority cannot be verified, even with the continuation of treatment (ie, inefficacy suspension), the independent monitoring committee will advise on the suspension of the study in order to prevent the continuation of treatment in the disadvantaged treatment group. In order to ensure an α error of 10% in the overall trial, the Lan and DeMets α spending function will be used to adjust for the redundancy of inspections in the intermediate and primary analyses, and the statistical significance of primary endpoint differences between groups will be studied. The O’Brien and Fleming-type α spending function will be used. A Bayesian predictive power based on a noninformative prior distribution will be used for inefficacy suspension determination in the proposed study.

#### Methods for Additional Analysis

Additional analyses will be conducted after the completion of the additional period and the finalization of the cases, from which data are obtained and presented in a statistical report for the representative physician.

#### Methods for Handling Missing Data

Questionable issues will be resolved after discussion between the representative physician and the statistical analysis manager. Missing values are not to be filled out, and details on the handling of outliers and abnormal values are to be regulated in the statistical analysis plan.

### Oversight and Monitoring

Quality control for ensuring compliance will be performed through monitoring by the Central Coordinating Unit of the Clinical Research Promotion Center at the Tokyo University Hospital. A monitoring plan has been created and approved by the representative physician and the authorizing clinical study examination committee. The principal physician who receives the monitoring reports is to notify the representative physician about the applicable report content as necessary.

No audit has been scheduled, and audits will only be conducted if serious problems are identified in the monitoring reports or if there are any events that impact patient safety.

All documents relating to the research are directly accessible for inspection.

### Composition of the Data Monitoring Committee

An independent Data and Safety Monitoring Board (DSMB) has been formed, and it consists of members with no financial or scientific conflicts of interest in this study. The DSMB chair is a clinician with extensive experience in respiratory medicine, DSMBs, and clinical research. The DSMB statistician is an experienced statistician. The other member of the DSMB is an experienced respiratory clinician. The committee’s remit is to protect the safety of trial participants by monitoring intermediate safety and operational data during trial implementation, and to provide suitable advice and suggestions to ensure the ethical and scientific validity of the study.

### Dissemination Plans

At 1 year after trial registration, the representative physician will report on the progress of this randomized controlled trial to the manager of the implementing medical institution and the Ministry of Health, Labor, and Welfare. A comprehensive report will be submitted at the end of the study.

Trial findings will be communicated at national and international scientific meetings by publications in a scientific journal. In addition, results will be disseminated to trial participants, study staff, clinicians, and patient groups via direct approaches and a variety of traditional and electronic media, including newsletters.

### Compensation Criteria

The principal physician, subphysician, and implementing medical institution must conduct suitable diagnoses and treatment and provide clinical care and other measures to ensure that the research subject is receiving the necessary care.

The representative physician has been enrolled in a clinical research insurance plan that includes compensation for the death or physical impediment of the research subject and medical expenses and care needed for the treatment of the health hazards in the research subject, in accordance with the payment conditions of clinical research insurance. The principal physician and subphysicians are also enrolled in a medical professional liability insurance plan.

## Results

The first trial participant was enrolled on April 3, 2020. Recruitment and registration are expected to be completed on September 30, 2020, while the follow-up of all participants is expected to be completed by October 31, 2020.

## Discussion

Preclinical data [12] have shown that ciclesonide can suppress the viral replication of coronaviruses in cell cultures [12,13], suggesting that ciclesonide can potentially be used to treat COVID-19. This multicenter, prospective, open-label randomized controlled trial has been designed to determine the efficacy and safety of ciclesonide in treating patients with asymptomatic and mild COVID-19. The rationale for choosing such patients was based on the findings of Iwabuchi et al [14,15], who indicated that the ideal ciclesonide administration period should be in the early intermediate stage of infection or the initial stage of pneumonia, prior to the worsening of symptoms.

The eligibility criteria in this study were designed to enroll study participants who had been diagnosed with SARS-CoV-2 infection without pneumonia and were not on any concomitant or prior medication that could affect the efficacy assessments. The trial processes of this study have been designed to be integrated within medical care at the participating hospitals, thereby enabling physicians to easily enroll participants without the need for a large research staff to compliment and implement the treatment, and learn more about the disease. Most of the clinical data will be routinely collected, except for SARS-CoV-2 antibody and viral genome quantification data. Trial staff have also been trained to adhere to the protocol.

There are some limitations that need to be considered. First, a placebo is not available because of the rights to the drug. Second, since this is an open-label study, there is potential for bias in the assessment. To avoid this, the primary endpoint analysis will be performed by a blinded independent radiologist. Third, ciclesonide is a steroid, and it is difficult to distinguish between its antiviral and anti-inflammatory effects.

Other trials that involve ciclesonide are in progress or in planning. Covis Pharma has launched a phase III clinical trial in the United States to evaluate the asthma drug, ciclesonide (ALVESCO), in treating nonhospitalized patients with symptomatic COVID-19 aged ≥12 years. In this US trial, participants will be given 320 µg of ciclesonide (ALVESCO) via metered-dose inhaler twice daily, along with standard supportive care or a placebo and standard supportive care. The primary endpoint of the Covis Pharma trial is the percentage of patients who were admitted to a hospital or died by day 30 [16]. The accumulation of findings from the RACCO study together with those from the other trials will be significant in developing interventions and treatments for COVID-19.

In conclusion, the RACCO study is an ongoing open-label randomized controlled trial that will provide the most definitive comparative effectiveness data to date and other important clinical outcomes data from the ciclesonide and symptomatic treatment groups. If the hypotheses that pneumonia exacerbation rate reduction is more significant in the ciclesonide treatment group than in the symptomatic treatment group and that ciclesonide is safe for use are valid, ciclesonide will provide an important therapeutic option for patients with COVID-19 that can be rapidly implemented in clinical practice.

## Abbreviations

CT: computed tomography
DSMB: Data and Safety Monitoring Board
EDC: electronic data capture
GCP: good clinical practice
jRCT: Japan Registry of Clinical Trials
MMRM: mixed model for repeated measures
PCR: polymerase chain reaction
RACCO: Randomized Ciclesonide COVID-19
REDCap: Research Electronic Data Capture

## References

[1] Wang C, Horby PW, Hayden FG, Gao GF (2020). A novel coronavirus outbreak of global health concern. Lancet.

[2] Dong E, Du H, Gardner L (2020). An interactive web-based dashboard to track COVID-19 in real time. Lancet Infect Dis.

[3] (2020). Archived: WHO Timeline - COVID-19. World Health Organization.

[4] Guan WJ, Ni ZY, Hu Y, Liang WH, Ou CQ, He JX, Liu L, Shan H, Lei CL, Hui DSC, Du B, Li LJ, Zeng G, Yuen KY, Chen RC, Tang CL, Wang T, Chen PY, Xiang J, Li SY, Wang JL, Liang ZJ, Peng YX, Wei L, Liu Y, Hu YH, Peng P, Wang JM, Liu JY, Chen Z, Li G, Zheng ZJ, Qiu SQ, Luo J, Ye CJ, Zhu SY, Zhong NS, China Medical Treatment Expert Group for Covid-19 (2020). Clinical Characteristics of Coronavirus Disease 2019 in China. N Engl J Med.

[5] Li LQ, Huang T, Wang YQ, Wang ZP, Liang Y, Huang TB, Zhang HY, Sun W, Wang Y (2020). COVID-19 patients' clinical characteristics, discharge rate, and fatality rate of meta-analysis. J Med Virol.

[6] (2020). Global Covid-19 Case Fatality Rates. The Centre for Evidence-Based Medicine.

[7] Rodriguez-Morales AJ, Cardona-Ospina JA, Gutiérrez-Ocampo E, Villamizar-Peña R, Holguin-Rivera Y, Escalera-Antezana JP, Alvarado-Arnez LE, Bonilla-Aldana DK, Franco-Paredes C, Henao-Martinez AF, Paniz-Mondolfi A, Lagos-Grisales GJ, Ramírez-Vallejo E, Suárez JA, Zambrano LI, Villamil-Gómez WE, Balbin-Ramon GJ, Rabaan AA, Harapan H, Dhama K, Nishiura H, Kataoka H, Ahmad T, Sah R, Latin American Network of Coronavirus Disease 2019-COVID-19 Research (LANCOVID-19) (2020). Clinical, laboratory and imaging features of COVID-19: A systematic review and meta-analysis. Travel Med Infect Dis.

[8] Chakraborty C, Sharma AR, Sharma G, Bhattacharya M, Lee SS (2020). SARS-CoV-2 causing pneumonia-associated respiratory disorder (COVID-19): diagnostic and proposed therapeutic options. Eur Rev Med Pharmacol Sci.

[9] Lu H (2020). Drug treatment options for the 2019-new coronavirus (2019-nCoV). Biosci Trends.

[10] Ciclesonide. Adis Insight.

[11] Schaffner TJ, Skoner DP (2009). Ciclesonide: a safe and effective inhaled corticosteroid for the treatment of asthma. J Asthma Allergy.

[12] Matsuyama S, Kawase M, Nao N, Shirato K, Ujike M, Kamitani W, Shimojima M, Fukushi S The inhaled corticosteroid ciclesonide blocks coronavirus RNA replication by targeting viral NSP15. bioRxiv.

[13] Meehyun K, Chang SY, Byun SY, Ianevski A, Choi I, d’Alexandry d’Orengiani ALPH, Kainov DE, Shum D, Min JY, Windisch MP Screening of FDA-approved drugs using a MERS-CoV clinical isolate from South Korea identifies potential therapeutic options for COVID-19. bioRxiv.

[14] Iwabuchi K, Koichiro Y, Yuichi K, Kota T, Yoshio K, Morishima T Improvements due to inhaled Ciclesonide in three patients in the early-mid pneumonia stages of COVID-19. Japanese Association for Infectious Diseases.

[15] Iwabuchi K, Yoshie K, Kurakami Y, Takahashi K, Kato Y, Morishima T (2020). Therapeutic potential of ciclesonide inahalation for COVID-19 pneumonia: Report of three cases. J Infect Chemother.

[16] A Study of the Safety and Efficacy of Ciclesonide in the Treatment of Non-hospitalized COVID-19 Patients. U.S. National Library of Medicine.

